# Ewing's Sarcoma as a Second Malignancy in Long-Term Survivors of Childhood Hematologic Malignancies

**DOI:** 10.1155/2016/5043640

**Published:** 2016-07-25

**Authors:** Fabian Wolpert, Michael A. Grotzer, Felix Niggli, Dieter Zimmermann, Elisabeth Rushing, Beata Bode-Lesniewska

**Affiliations:** ^1^Department of Neurology, University Hospital Zurich, Frauenklinikstrasse 26, 8091 Zurich, Switzerland; ^2^Department of Medical Oncology, University Hospital Zurich, Rämistrasse 100, 8091 Zurich, Switzerland; ^3^Department of Pediatric Oncology, University Children's Hospital, Zurich, Steinwiesstrasse 75, 8032 Zurich, Switzerland; ^4^Institute of Surgical Pathology, University Hospital Zurich, Schmelzbergstrasse 12, 8091 Zurich, Switzerland; ^5^Department of Neuropathology, University Hospital Zurich, Schmelzbergstrasse 12, 8091 Zurich, Switzerland

## Abstract

Modern multimodal treatment has significantly increased survival for patients affected by hematologic malignancies, especially in childhood. Following remission, however, the risk of developing a further malignancy is an important issue. The long-term estimated risk of developing a sarcoma as a secondary malignancy is increased severalfold in comparison to the general population. Ewing's sarcoma family encompasses a group of highly aggressive, undifferentiated, intra- and extraosseous, mesenchymal tumors, caused by several types of translocations usually involving the* EWSR1* gene. Translocation associated sarcomas, such as Ewing sarcoma, are only rarely encountered as therapy associated secondary tumors. We describe the clinical course and management of three patients from a single institution with Ewing's sarcoma that followed successfully treated lymphoblastic T-cell leukemia or non-Hodgkin lymphoma. The literature on secondary Ewing's sarcoma is summarized and possible pathogenic mechanisms are critically discussed.

## 1. Introduction

Modern multimodal treatment strategies have significantly increased the survival of patients affected by hematologic malignancies, including indolent and aggressive forms of lymphoma and leukemia [1, 2]. In long-term survivors of childhood cancer, the risk of a second cancer is an important issue [3]. Standard treatment for newly diagnosed malignancies varies according to the phenotype, the line of differentiation, biological markers, and extent of the disease and comprises various combinations of chemotherapy [4], radiotherapy, targeted therapy with monoclonal antibodies, and stem cell transplantation, all of which have been implicated as risk factors for second malignancies. The most common second cancers reported in this population include cancers of the head and neck, melanoma, lung cancer, colon cancer, bladder cancer, renal cancer, Hodgkin disease, leukemia, and Kaposi sarcoma [5]. Patients who received radiation therapy are more likely to develop sarcomas, breast cancers, and mesothelioma compared with patients who have not received it [5].

Ewing's sarcoma (ES) is an aggressive malignancy occurring as a primary tumor mostly in adolescents and young adults with an incidence of about 1/1 000 000, male to female ratio being 1,5 : 1 [6]. Most cases of ESs (80%) originate in bones, where this entity comprises the second most common primary tumor following osteosarcoma in children and adolescents. Up to 20% of primary ESs are located extraosseously in soft tissues, affecting not only extremities but also various internal organs [7–10]. ESs are characterized at the molecular level by the presence of a balanced translocation between the* EWSR1* gene and a member of the ETS gene family, in at least 80% of cases the partner gene being the* FLI1* gene (translocation t(11;22) (q24;q12)* (EWS-FLI1)*) [11]. This molecular signature and the clinical characteristics of the patients are typical for the peripheral primitive neuroectodermal tumor (PNET) as well. Accordingly, in the current WHO classification [11], PNET once considered a separate entity is now considered to represent part of the histologic spectrum of the “Ewing family of tumors” [6, 12]. The presence of the fusion gene containing the* EWSR1* gene and a member of the ETS family is the most important tumorigenic factor in ES; however, recent genomewide association studies (GWAS) have demonstrated an association of this tumor with the germline background of the affected patients [13]. These studies provide a plausible explanation for the epidemiological finding of a different incidence of ESs across human populations, for example, the significantly higher incidence in patients of Caucasian compared to African origin.

ESs, in contrast to sarcomas with complex karyotypes and without recurring genetic aberrations such as osteosarcoma, have only rarely been reported to occur secondary to cancer therapy, including multimodal therapy for hematologic malignancies [7, 14, 15]. The aim of this study is to analyze three cases of secondary ES in patients from our institutions with T-cell leukemia or non-Hodgkin lymphoma (NHL) as well as summarize documented similar cases from the literature. In addition, the literature review on Ewing's sarcoma as a secondary cancer is summarized and possible pathogenic mechanisms are critically discussed.

## 2. Materials and Methods

### 2.1. Patients

Three patients with a diagnosis of a genetically confirmed Ewing sarcoma who were previously treated for leukemia or lymphoma during the period of 1991 to 2014 were identified among 98 Ewing sarcoma patients in the archives of the Institute of Surgical Pathology, University Hospital Zurich, Switzerland. Complete follow-up data were available for all 3 patients, including information regarding tumor site and specific treatment modalities of the primary as well as of the secondary malignancy. The available histopathological slides of all 3 cases were reviewed to confirm the diagnosis of ES. A review of the literature published in PUBMED of previously reported cases of ES following another primary malignancy was performed.

The study has been carried out according to the ethical guidelines of our institution.

### 2.2. Histology and Immunohistochemistry

Tumor tissue samples were fixed in buffered 4% formalin and embedded in paraffin. 2 *μ*m thick sections were stained with hematoxylin and eosin (H&E) according to standard procedures. Immunohistochemistry (IHC) using the CD99 antibody (dilution 1 : 25, clone HO36-1.1, Novocastra Lab Ltd) was performed on 2 *μ*m thick paraffin sections, using the Ventana Benchmark XT automated staining system (Ventana Medical Systems, Tucson, Arizona).

### 2.3. Fluorescence In Situ Hybridization (FISH)

Fluorescence in situ hybridization (FISH) studies were performed on formalin-fixed, paraffin-embedded, 4 *μ*m thick tissue sections as described earlier [16]. Dual color break-apart FISH detecting translocations involving the* EWSR1* (22q11) gene was performed using commercial probes (Vysis, Abbott AG, Baar, Switzerland). The SpectrumOrange- and SpectrumGreen-labeled EWSR1 probes covered 500 kb and 1,100 kb proximal and distal to the EWSR1 gene. The fluorescence staining was visualized with an Olympus BX61 microscope (Olympus, Volketswil, Switzerland) equipped with DAPI, SpectrumGreen and SpectrumOrange filters. Images were acquired with a CCD camera and processed with the AnalySIS imaging software (Soft Imagining System, Munster, Germany). For the assessment of* EWSR1* gene rearrangements at least 50 nonoverlapping tumor nuclei were analyzed. If the sample contained at least 25% of split red and green signals, the tumor was regarded as translocation positive. The signal was considered as split, when red and green fluorescent spots were separated by at least twice the distance occupied by a single probe [17].

### 2.4. RNA Extraction and RT-PCR

To detect EWSR1-FLI1 and EWSR1-ERG fusions transcripts, total RNA was extracted form paraffin-embedded tissue as described earlier [18]. Amplification was done in a GeneAmp PCR System 9700 (Applied Biosystems) using an OneStep RT-PCR Kit (Qiagen). The 25 *μ*L reactions contained 0.4 mM dNTP, 1x OneStep RT-PCR Buffer, 5 *μ*L Q-solution, 1 *μ*L enzyme mix (all from Qiagen), and 8 units of RNase OUT (Life Technologies). The primer pairs were as follows: (A) TCCTACAGCCAA GCTCCAAGTC (EWSR1-Ex7b) and ATTCATGTTGGGCTTGCTTTTC (FLI1-Ex9); (B) AGAGTAGCTATGGTCAACAA (EWSR1-Ex7a) and CCCA(TA)GCTCCTCT TCTG (FLI1-Ex6); (C) TCCTACAGCCAAGCTCCAAGTC (EWSR1-Ex7b) and CTCCAGGAGGAACTGCCAAA (ERG-Ex9).

The reverse transcription was done for 30 minutes at 50°C, followed by a PCR activation step for 15 minutes at 95°C and 40 cycles consisting of 1 minute at 94°C, 1 minute at 56°C, and 1 minute at 72°C followed by final extension at 72°C for 10 minutes. RNA-integrity was tested by running ACTB- and IPO8-specific internal control reactions for each sample.

## 3. Results

### 3.1. Patients

The basic patient characteristics are summarized in Table 1.

#### 3.1.1. Patient 1

**Figure 1:**
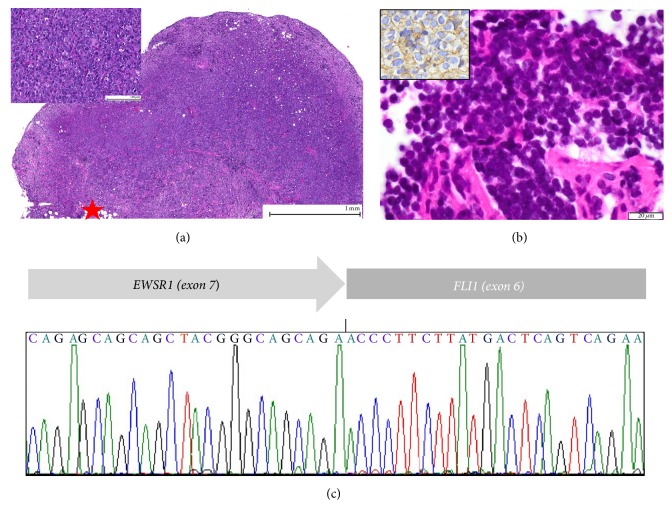
(a) Biopsy of the cervical mass of patient 1, 3 years prior to current presentation. Diffuse infiltration of the lymph node tissue with effacement of the lymph node architecture and spread to the adjacent adipose tissue (star) by sheets of atypical lymphatic blasts (inset) (H&E; original magnification 400x). (b) Core biopsy of the paravertebral mass of patient 1. Solid small, blue round undifferentiated tumor with strong immunohistochemical membranous expression of CD99 (brown reaction product shown in the inset) (H&E; original magnification 400x). (c) Sanger-sequencing of the RT-PCR amplicon derived from the fusion transcript reveals the most common EWSR1-FLI1 fusion of exon 7 to exon 6, respectively, in the paravertebral tumor tissue of patient 1 confirming the diagnosis of a Ewing sarcoma.

T-cell lymphoblastic non-Hodgkin lymphoma was diagnosed in this male patient at the age of 5 years. He first presented with supraclavicular swelling. Histopathologic examination of the resected lymph node revealed high grade T-cell lymphoblastic non-Hodgkin lymphoma (Figure 1(a); unfortunately, except for the text of the final diagnosis and one H&E slide no further histopathological details are available). Diagnostic workup including chest and abdominal CT scans revealed no further tumor manifestations. He was treated according to the NHL-BFM 90 therapy (Berlin-Frankfurt-Munster), which comprises cyclophosphamide, dexamethasone, ifosfamide, methotrexate, cytarabine, prednisolone, etoposide, and cyclophosphamide without radiotherapy. Chemotherapy was completed after 2.5 years in 03/1998. The patient showed complete remission in the comprehensive follow-up investigations that included laboratory testing, CT scan of brain, chest, and abdomen, bone scan, bone marrow biopsy, cardiac scan, and renal scan.

Three years after the first presentation, he developed nocturnal thigh pain. A bone scan showed a large paravertebral (level L5) mass. Chest and thorax CT revealed lung metastases. Biopsy of the paravertebral lesion showed a malignant small, blue, round, cell tumor, histopathologically consistent with ES (Figure 1(b)). The diagnosis was confirmed by RT-PCR demonstrating the t(11;22) translocation with the* EWSR1/FLI1* fusion transcript (Figure 1(c)).

The patient received chemotherapy with vincristine, actinomycin, ifosfamide, adriamycin, carboplatin, epirubicin, and etoposide, based on the CWS-96 protocol (high risk) [19]. Furthermore, he received radiotherapy to the chest and paravertebral region with a cumulative dose of 44.8 Gy. Subsequently, CT scans of the chest, abdomen, and spine revealed complete remission of the pulmonary metastases and size reduction of the left sided paravertebral mass at L4-5 level.

One year later, the patient underwent high dose chemotherapy with busulfan and alkeran and autologous stem cell transplantation. Unfortunately, he developed an infection with an unknown pathogen one month later that proved refractory to antibiotic treatment and he died from acute respiratory distress syndrome.

#### 3.1.2. Patient 2

Acute lymphoblastic T-cell leukemia was diagnosed in this male patient at the age of 16 years. He presented with a mediastinal mass without central nervous system involvement. Bone marrow aspiration showed 95% blasts infiltration: CD3c+; CD3+; CD4+; CD8+; CD7+; CD5+; CD34+; CD56+; CD2+; TDT+; TCR a/b+. The patient underwent chemotherapy with vincristine, daunorubicin, L-asparaginase, methotrexate (MTX), and Ara-C. Dexamethasone and thioguanine were given during induction and reinduction. He received whole brain radiation (cumulative dose 12 Gy) and 4 doses of intrathecal therapy with MTX, prednisolone, and Ara-C. After completion of therapy in 10/2002, the patient showed hematological remission in the follow-up controls.

**Figure 2:**
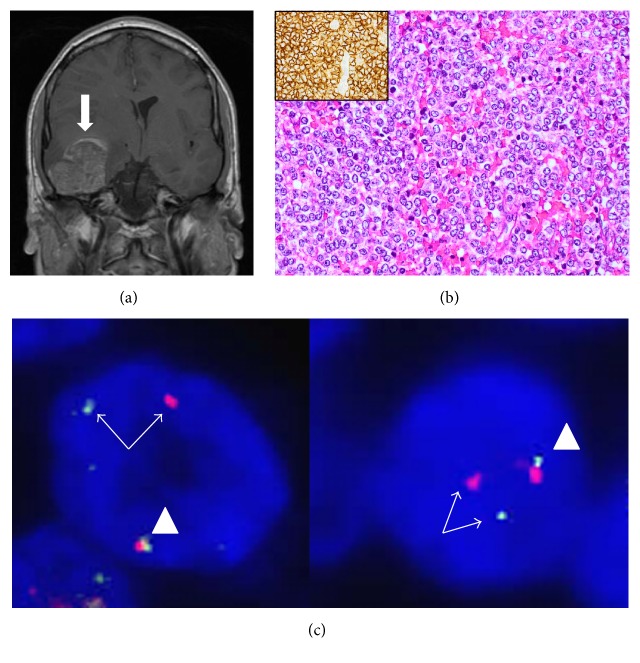
(a) Coronary T1 MRI section demonstrating a 4 cm mass (arrow) within the right temporal lobe with middle line shift in patient 2. (b) Biopsy of the intracerebral temporal tumor of patient 2 shows solid small, blue round undifferentiated proliferation with a strong immunohistochemical membranous expression of CD99 (brown reaction product shown in the inset) (H&E; original magnification 200x). (c) Fluorescence in situ hybridization (FISH) of the brain tumor of patient 2, demonstrating the rearrangement of the EWSR1 gene. The nuclei of the tumor cells contain one fused signal (arrow head) and one pair of split green and red signals (arrows).

Five years and 8 months later, the patient presented to the emergency unit with headache and vomiting. MRI imaging revealed a large intracerebral tumor mass (Figure 2(a)). Resection of the tumor revealed a strongly CD99 positive, small, blue, round cell malignant tumor on histopathological examination (Figure 2(b)) that lacked the expression of lymphatic markers. No rearrangement of the TCR-*γ* gene locus was found on genotyping. The RT-PCR of the fusion products of the two most common Ewing sarcoma translocations t(11;22)* EWSR1/FLI1* and t(21;22)* ESWR1/ERG* was negative; however, FISH analysis revealed the* EWSR1* gene rearrangement (Figure 2(c)), confirming the diagnosis of Ewing sarcoma and suggesting the presence of one of the rare ES translocation types.

Postoperatively, radiotherapy with 54 Gy was applied to the right temporoparietal region over 6 weeks. The patient underwent two cycles of cisplatin and CCNU/lomustine; however, chemotherapy had to be discontinued in 02/2007 due to myelotoxicity. At the most recent follow-up in 7/2015, the patient was alive and doing well under anticonvulsive treatment; however, a small mass was found on control MRI in the right frontal lobe, suggesting a metastasis.

#### 3.1.3. Patient 3

This female patient developed acute T-cell leukemia at the age of 9 years. She initially presented with fatigue accompanied by leukocytosis, mediastinal mass, and hepatosplenomegaly, but without CNS involvement. Cytogenetic analysis of the bone marrow aspiration revealed t(1;16), loss of TLX3, and biallelic deletion of 12p. The patient was treated according to the high risk protocol of the ALL-BFM-2000 trial with prednisone, vincristine, daunorubicin, L-asparaginase, cyclophosphamide, high dose methotrexate, cytarabine, mercaptopurine, thioguanine, and etoposide. Due to the risk stratification an allogenic bone marrow transplantation with an unrelated donor had to be performed five months after the presentation. Conditioning regimen included total body irradiation with a cumulative dose of 12 Gy (6 × 2 Gy), etoposide, and antithymocyte globulin Fresenius. A graft versus host disease- (GvHD-) prophylaxis with MTX and cyclosporin A was applied. In the following controls, she showed an enduring remission.

**Figure 3:**
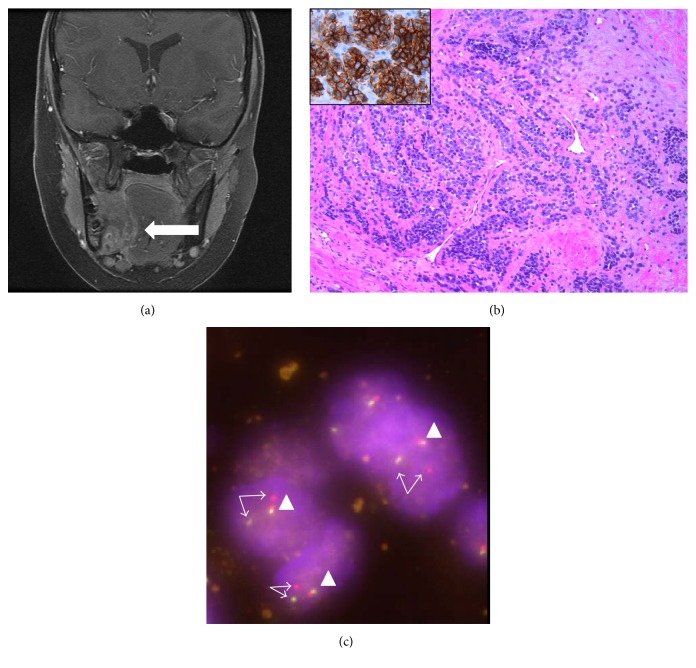
(a) Coronary T1 MRI section demonstrating a large mass (arrow) of the right mandible with infiltration and displacement of the tongue of patient 3. (b) Biopsy of the intraoral mass of patient 3. Diffuse infiltrates of a small, blue round, undifferentiated tumor with strong immunohistochemical membranous expression of CD99 (brown reaction product shown in the inset) (H&E; original magnification 100x). (c) Fluorescence in situ hybridization (FISH) of the jaw tumor of patient 3, demonstrating the rearrangement of the EWSR1 gene. The nuclei of the tumor cells contain one fused signal (arrow head) and one pair of split green and red signals (arrows).

Two years and four months later, the patient reported a swelling in the region of the right 2nd lower molar tooth. MRI of the head (Figure 3(a)) demonstrated a large tumor mass of the right lower jaw that infiltrated the soft tissues of the floor of the mouth and displaced the tongue. Biopsy revealed the diagnosis of Ewing sarcoma with characteristic small, round, and blue cell histology and typical strong and membranous immunohistochemical expression of CD99 protein (Figure 3(b)). This diagnosis was confirmed by the demonstration of the* EWSR1* gene rearrangement on FISH analysis (Figure 3(c)), with the fusion transcript* EWSR1/FLI1* of the diagnostic translocation by RT-PCR (the same type of the translocation as in patient 1) (Figure 1(c)). The patient was then treated according to the Euro-Ewing 99 protocol with vincristine, ifosfamide, doxorubicin, etoposide, actinomycin D, and cyclophosphamide. Local tumor control was achieved with total tumor resection and mandibular reconstruction with a fibula plastic. Unfortunately, local ES recurrence with biopsy confirmation was detected one year later and the patient underwent radiotherapy with a cumulative dose of 54 Gy (30 × 1,8 Gy) accompanied by second-line chemotherapy (cyclophosphamide, topotecan). Bone metastases in the right femur and fibula were first diagnosed in the following year, followed by wide metastatic spread (spine, lung), which prompted renewed chemotherapy with ifosfamide and palliative mediastinal radiotherapy.

### 3.2. Review of the Literature

There are only few reports on ES as a secondary malignancy (Table 2), mostly comprising single case reports or short series describing ES following treatment for unrelated tumors, such as breast carcinoma [20, 21], retinoblastoma [22, 23], or testicular mixed germ cell tumor [24]. The largest series of secondary ES by Applebaum et al. [25] evaluated data from the Surveillance, Epidemiology, and End Results Program (SEER) database between 1973 and 2008 and found 58 cases of secondary ES, accounting for up to 2.1% of all ES. Only 12.1% of patients received radiation to the ES site, while the frequency of previous chemotherapy is not reported. Interestingly, five further patients were diagnosed with ES concurrent with other tumors and were therefore not included in the study group of “secondary” ES. This study, however, is purely epidemiological, not including reevaluation of the diagnoses or molecular confirmation of ES. The most common group of primary tumors did not comprise further specified carcinomas (41%: 24 of 58), with rare cases of non-ES sarcomas, melanomas, brain tumors, or other neoplasia as a the primary tumor. In 24% (14/58) of cases in this study, hematological malignancies preceded secondary ES, representing the second most common primary malignancy group after carcinomas.

The first case of genetically confirmed ES following a hematological malignancy (ALL) was described in 1984 by Tilly et al. [26], although the first case without genetic documentation was mentioned by Meadows et al. [27] in 1977. According to the review of available literature, 38 cases (including the current study) of secondary ES have been described following the diagnosis of a hematologic malignancy (Table 2); however, confirmatory data on genetic tumor background (karyotype, FISH, and/or RT-PCR) has been provided in only 14 cases [14, 26, 28–33]. 

The diagnosis of secondary ES followed in 1 to 16 years (mean 6 y) the diagnosis of a hematologic neoplasm, most often an acute lymphoblastic leukemia (46%: 11 of 24 cases with available information), Hodgkin lymphoma, or an aggressive form of non-Hodgkin lymphoma (5 cases (20%) each). There were only two patients with myelogenous neoplasia and 1 with no further specified small cell non-Hodgkin lymphoma. The age of the patients at the first tumor diagnosis ranged from 3 to 22 y (mean 10 y) and at ES diagnosis from 7 to 27 y (mean 16 y), with only one substantially older patient (diagnoses at 57 and 65 y, resp.). All patients with available data received disease specific chemotherapy for their primary malignancy, consisting of a combination of various groups of cytostatic and cytotoxic substances (Table 2) with no obvious causative association. Secondary ES occurred most commonly in the chest wall and long bones (7 cases each) followed by visceral organs (3), pelvis (2), somatic soft tissue (2), and mandible (1).

## 4. Discussion

Modern multimodal therapy has led to markedly improved long-term survival of cancer patients. Concomitantly, a well-documented increased incidence of malignancies in cancer survivors in comparison to general population has been observed. Most cases of secondary tumors can be attributed to the effects of long-term toxicity of radio- and/or chemotherapy; however, the underlying mechanisms of tumorigenesis in this setting remain unclear. In most instances, nonrandom somatic molecular or chromosomal alterations are thought to be involved in the pathogenesis of secondary tumors. Among sarcomas, most therapy associated tumors belong to the category of tumors showing complex karyotypes with mostly nonrecurring genetic aberrations, including undifferentiated pleomorphic sarcoma, osteosarcoma, or rarely malignant peripheral nerve sheath tumors [41]. Therapy associated angiosarcomas show recurrent amplifications of the c-myc gene [42]. Alternatively, sarcomas with simple karyotypes, carrying specific translocations or point mutations, have only rarely been described in the context of secondary, therapy associated tumors [43]. The underlying pathomechanism of therapy-induced malignancies has been elucidated for only selected secondary tumor entities. For example, secondary leukemia following the use of mitoxantrone as an immune modulating drug in patients with multiple sclerosis has been attributed to inhibition of topoisomerase II (TI-II) [44, 45]. TI-II inhibition has been identified as being crucial in the development of the t(15;17) balanced translocation and generation of the PML-RARalpha fusion transcript characteristic for promyelocytic leukemia [46]. Anthracyclines or other drugs inhibiting TI-II are part of therapeutic regimens for hematologic malignancies (Table 2); however, it remains to be elucidated whether these agents play pathogenetically relevant role in the occurrence of ES as a secondary tumor.

It is well known that immunodeficiency after radio-/chemotherapy and hematopoietic stem cell transplantation are major challenges for survivors of hematological malignancies [47, 48]. Impaired antitumor activity of the innate or adaptive immune system might represent critical factors that predispose to the failure of the system to detect and eliminate newly mutated cells carrying the Ewing sarcoma translocation, thus allowing for the initiation of secondary ES.

Two of the patients described in the current study received radiation therapy, one total body radiation (TBI) and one to the site of the ES occurrence (brain), suggesting a possible role for the radiotherapy in the induction of the subsequent ES. However, among the data in the literature, previous irradiation to the ES site was noted in only 12.1% of the patients in the study of Applebaum et al. [25] and among the cases compiled in Table 2 with available data on the previous radiation, there were some patients with TBI (2 of 7), but also patients with no radiation to the body parts later affected by ES.

The pathogenesis of Ewing sarcoma is strongly linked to the presence of a translocation between the* EWSR1* gene and a member of the ETS gene family [11]. The occurrence of the translocation itself was for a long time considered to be a random event. In addition, the risk of developing ES has been linked to increased maternal age [44] and there are associations with exposure to environmental toxins [3, 8, 9]. Alternatively, there are striking differences in the incidence ES according to racial background, ES showing significantly higher incidence among Caucasians than in patients of African origin. Recently published observations [13] of a different frequency of SNPs variants of the* EGR2* gene related to Ewing sarcoma susceptibility and may account for this observation, since the frequency of the SNP variant is significantly higher in the non-Africans than in patients of African origin. This important study provides deeper insights into the etiology of the occurrence of this specific translocation associated sarcoma and allows the conclusion that this is not a random disease but rather the expression of a germ line predisposition syndrome, thus accounting for the strong predilection of the Ewing sarcoma to occur in young patients, similar to other genetically linked tumors.

Due to advances in next generation sequencing techniques and the application of powerful computational tools, similar germ line association studies (genomewide association studies, GWAS) are currently possible on large sets of genetic data for many tumor types. Germ line associations of patients with ALL has been recorded for susceptibility, drug response, and toxicities of ALL therapy [49]. Speculatively, as yet not identified common genetic predisposition trait may exist for Ewing sarcoma and some other neoplasia, including hematologic diseases.

Interestingly, in the recent comprehensive study of germ line mutations in the best characterized genes associated with cancer predisposition syndromes in pediatric cancer patients, 5 patients with relevant mutations (3 patients with TP53 gene mutations, one patients with PMS2 gene mutation, and one patient with RET gene mutation) were identified among 46 (10.8%) patients with Ewing sarcoma, frequency of which was higher than in cases of rhabdomyosarcoma (7%, 3/43) [50]. Ewing sarcoma has been rarely reported in well-defined germ line mutation associated syndromes (Cowden syndrome [51], multiple osteochondromatosis [52], and Williams syndrome [15]); however it has never been listed as a syndrome defining entity. Of note, none of the patients in our study had known cancer predisposition syndromes or family history of a recognized risk constellation.

According to Sultan et al. [53], 3% of Ewing sarcoma patients develop secondary tumors (4–9-fold risk compared to the general population, the risk being higher for children). In such cases, therapy associated hematologic neoplasms, radiation associated sarcomas, or carcinomas follow multimodal Ewing sarcoma therapy. The relative risk of developing a second malignancy seems to be higher for Ewing sarcoma patients in comparison to other tumors (except for retinoblastoma and primitive neuroectodermal tumors of the central nervous system), suggesting a relationship of ES to either aggressive chemotherapy or genetic predisposition, which may be augmented by the application of cytostatic and cytotoxic drugs.

In summary, we provide a detailed description of well-documented cases of Ewing sarcoma following a primary hematologic malignancy. Additionally we have reviewed the literature on this association and discussed the possible pathogenetic aspects. As the survival of cancer patients will continue to improve in the future, it is important to understand the risks associated with this success, including the risk of the development of second malignancies, both therapy related and/or linked to the genetic background of the patients.

## Figures and Tables

**Table 1:** Clinical data on the patients in the current study.

Pat.	Gender	Age at Dx (T-ALL)	Age at Dx (ES)	Latency (y)	Location of ES	Treatment of the primary hematological malignancy				
						Anthracyclines, TPII-Inh	Vinca	Alkylating agent	Antimetabolite	RT
1	m	5	8	3	Paravertebral (L5)	ADR	None	CPA, IFO	Ara-C	None
2	m	16	21	5	Brain, temporal lobe	DNR	VCR	CPA	MTX, Ara-C, 6-MP, 6-TG	Whole brain (12 Gy)
3	f	9	11	2	Mandible	DNR	VCR	CPA	MTX, Ara-C, 6-MP, 6-TG	Whole body (12 Gy)

DNR, daunorubicin; ADR, doxorubicin; VCR, vincristine; CPA, cyclophosphamide; IFO, ifosfamide; MTX, methotrexate; Ara-C, cytarabine; 6-MP, 6-mercaptopurine; 6-TG, thioguanine; RT, radiotherapy.

**Table 2:** Review of the published cases of the Ewing sarcoma following the therapy of the hematologic malignancy as a primary tumor.

Number	Nr with genetic confirmation (any method)	Reference	Pub year	Age at Dx PT	Age at Dx ES	Latency (y)	Primary cancer	Location ES	Molecular test	Treatment of the primary hematological malignancy						
										Anthracyclines, TopoII-INH	Vinca	Alkylating agent	Antimetabolite	Other drugs	Rth	Additional treatment
1		Meadows et al. [27]	1977			5	ALL	ND	ND							
2	1	Tilly et al. [26]	1984	8	19	11	ALL	Sacrum	Karyotype	DNR	VCR	DTIC		PD	Cranial RT 24 Gy	
3		Anselmo et al. [34]	1994	20	27	7	HL	Chest wall	ND							
4	2	Zoubek et al. [28]	1995	12	20	8	Anaplastic T LCL	Pelvis	Karyotype RT-PCR	VP16		CPA, MEL	MTX, Ara-C		TBI 12 Gy	SCT (auto)
5		Delgado-Chavez et al. [35]	1995	22	26	4	HL	Chest wall	ND							
6	3	Antillon et al. [29]	1997	7	16	9	ALL	Tibia	RT-PCR	DNR	VCR			PD, L-asp		
7		Aparicio et al. [30]	1998	3	15	12	ALL	Ulna	ND	ADR	VCR		6-MP, MTX, Ara-C	L-asp	Cranial RT	
8				11	18	7	AML	Fibula	ND	DNR, ADR, MTZ, VP16			6-MP, 6-TG, Ara-C			
9		Bisogno et al. [31]	2004	4	7	3	HL	Thoracic wall	ND	ADR	VBL	DTIC			RT 20 Gy	
10				7	18	11	CML	Thigh	ND				HU		TBI 9 Gy,	SCT (allo)
11		Suarez et al. [36]	2004	8	9	1	ALL	Chest wall	ND	ARD	VCR	CPA	MTX, 6-MP, Ara-C	PD		
12	4	Kim et al. [32]	2005	3	7	4	ALL	Humerus	Karyotype RT-PCR	ADR	VCR	CPA	6-TG, MTX, Ara-C	PD, L-asp		
13	5	Spunt et al. [33]	2006	9	12	3	LCL	Rib	FISH	ADR	VCR	CPA		PD	RT 15 Gy	
14	6			8	16	8	ALL	Tibia	RT-PCR	DNR, VP16	VCR	CPA	6-MP, MTX, Ara-C	PD, L-asp		
15	7			7	23	16	Small, noncleaved cell lymphoma	Rib	FISH	ADR	VCR	CPA	MTX, Ara-C			
16	8			12	17	5	HL	Chest wall	RT-PCR	ADR, BLE	VBL		MTX	PD		
17		Khadwal et al. [37]	2006	8	10	2	HL	Knee	ND	ADR, BLE	VBL				RT 24 Gy	
18	9	Osone et al. [38]	2007	10	16	6	ALL	Urinary bladder	RT-PCR	ADR	VCR	CPA	MTX, 6-TG	PD, L-asp	none	
19		Renard et al. [39]	2011	7	19	12	ALL	ND	ND	ND	ND	ND	ND	ND	ND	ND
20	10	Lim et al. [40]	2013	20	22	2	Burkitt	Adrenal gland	FISH	ADR	VCR	CPA	MTX, Ara-C	DEXA		
21	11	Hiramoto et al. [14]	2013	57	65	8	B-LCL	Soft tissue back	RT-PCR	DNR	VCR	CPA		PD		GCSF
14 more cases		Applebaum et al. [25]	2013	ND	ND	ND	ND	ND	ND	ND	ND	ND	ND	ND	ND	ND
36	12	Current series	2016	5	8	3	Lymphoblastic lymphoma	Paravertebral	RT-PCR	ADR		CPA, IFO	Ara-C			
37	13	Current series	2016	16	21	4	T-ALL	Brain	FISH	DNR	VCR	CPA	MTX, Ara-C, 6-MP, 6-TG		RT 12 Gy	
38	14	Current series	2016	9	11	2	T-ALL	Mandible	FISH, RT-PCR	DNR	VCR	CPA	MTX, Ara-C, 6-MP		TBI RT 12 Gy	

DNR, daunorubicin; VP-16, etoposide; ADR, doxorubicin; ACT, actinomycin D; MTZ, mitoxantrone; BLE, bleomycin; VCR, vincristine; VBL, vinblastine; DTIC, dacarbazine; CPA, cyclophosphamide; MEL, melphalan; IFO, ifosfamide; MTX, methotrexate; Ara-C, cytarabine; 6-MP, 6-mercaptopurine; 6-TG, thioguanine; HU, hydroxyurea; PD, prednisone; L-asp, L-asparaginase; RT, radiotherapy; TBI, total body irradiation; SCT, stem cell transplantation. ND: no data.
